# Practice patterns in prepectoral implant-based breast reconstruction: A survey of UK plastic surgeons^✰^

**DOI:** 10.1016/j.jpra.2026.08.015

**Published:** 2026-08-19

**Authors:** Krzysztof Sosnowski, Alessandra Lim, David Kwan Ryung Lee, Chloe Jordan, Rushabh Shah, Charles M. Malata

**Affiliations:** a School of Clinical Medicine, University of Cambridge, Cambridge, United Kingdom; b Plastic Surgery Department, Cambridge University Hospitals NHS Foundation Trust, Addenbrooke’s Hospital, Cambridge, UK; c Cambridge Breast Unit, Addenbrooke’s Hospital, Cambridge University Hospitals NHS Foundation Trust, Cambridge, United Kingdom; d Anglia Ruskin University School of Medicine, Chelmsford, Cambridge, United Kingdom

**Keywords:** Implant breast reconstruction, Prepectoral reconstruction, National survey, Plastic surgeons, Acellular dermal matrix

## Abstract

**Background:**

Prepectoral prosthetic breast reconstruction following mastectomy is an increasingly adopted alternative to the subpectoral. Despite its growing use, contemporary UK data specifically examining pre-pectoral practice among plastic surgeons remains limited. Variation also exists in surgical technique, acellular dermal matrix (ADM) use and clinical decision-making.

**Methods:**

A national cross-sectional online survey was distributed to UK consultant plastic surgeons performing breast reconstruction within the NHS. The survey captured geographical distribution, annual reconstruction volumes, frequency of prepectoral implant use, ADM preferences, implant coverage technique and factors influencing clinical decision-making. Descriptive statistics, Friedman’s test and pairwise Wilcoxon signed-rank tests with Bonferroni adjustment were employed for statistical analysis.

**Results:**

Twenty UK plastic surgeons responded. Of these, 80% (n = 16) reported performing pre-pectoral reconstruction. Among all respondents, 75% (n = 15) reported using the pre-pectoral plane in at least 50.0% of their implant-based reconstructions. Soft tissue coverage was rated the most important factor influencing plane selection, and was ranked significantly higher than smoking status (*p* = 0.01). Porcine ADM was used by 14 of 16 respondents (87.5%), and ADM availability was the only factor reaching significance on pairwise testing for product selection (*p* = 0.022). Complete anterior-posterior implant coverage was used by 10 of 16 surgeons (62.5%). Free-text responses suggested a perceived reduction in implant-based reconstruction within plastic surgery practice, with increasing involvement of breast and oncoplastic surgeons.

**Conclusion:**

This survey identified variation in pre-pectoral implant-based breast reconstruction practice among responding UK consultant plastic surgeons, particularly in relation to ADM selection and implant coverage technique. Porcine ADM predominated, and local availability appeared to influence product selection. Free-text responses raised relevant questions regarding changing patterns of service delivery, trainee exposure and outcome monitoring in implant-based breast reconstruction.

## Introduction

In recent decades, rates of implant-based breast reconstruction (IBBR) following mastectomy have increased.^1^^,^^2^ Breast reconstruction plays an important role in patient quality of life and psychological outcomes, supporting the need to optimise clinical practice.^3^ The aims of IBBR are to provide a safe and aesthetically acceptable reconstruction, while minimising complications, patient morbidity and treatment burden.^4^ The introduction of adjunctive materials, including acellular dermal matrices (ADM), has contributed to the evolution of implant-based techniques.^5^ One important technical development has been renewed interest in pre-pectoral implant placement, as an alternative to the more established subpectoral approach.

Pre-pectoral reconstruction involves placement of the implant in the subcutaneous plane, anterior to the pectoralis major muscle. By avoiding elevation and/or division of the pectoralis major, this technique preserves the native chest wall anatomy and may reduce morbidity associated with subpectoral reconstruction.^6^ Previous studies have suggested that pre-pectoral breast reconstruction may reduce animation deformity, defined by Fracol et al. as the aesthetically undesirable result of ‘implant displacement with pectoralis muscle contraction’ seen with subpectoral implants.^7^, ^8^, ^9^ ADM may also help to define the implant pocket, support implant position and provide additional implant coverage.^10^

However, the benefits of prepectoral reconstruction need to be balanced against its limitations. Prepectoral techniques have been associated with higher rates of rippling in some series, and several studies have shown no significant difference in patient satisfaction between subpectoral and prepectoral approaches.^11^^,^^12^ Evidence regarding capsular contracture is also mixed.^12^^,^^13^ As such, the decision to use a prepectoral pocket remains dependent on patient factors, mastectomy flap quality, soft tissue coverage, planned adjuvant treatment and surgeon preference.

Significant variation exists in the practice of prepectoral reconstruction.^14^ This includes variation in ADM or mesh selection, such as porcine, bovine, human-derived or synthetic products, as well as differences in implant coverage technique, including anterior coverage, complete anterior-posterior coverage and composite ADM techniques.^14^ In the absence of clear evidence favouring a single material or coverage strategy, understanding how surgeons make these decisions is clinically relevant.

Despite increasing use of prepectoral breast reconstruction, long-term comparative evidence remains limited and practice patterns continue to vary. Previous UK survey data have demonstrated heterogeneity in cosmetic breast implant selection, but contemporary data specifically examining postmastectomy pre-pectoral breast reconstruction practice among UK plastic surgeons remain limited.^15^ This survey therefore aimed to describe current reported practice among UK consultant plastic surgeons performing implant-based breast reconstruction in the NHS, with particular focus on factors influencing pre-pectoral reconstruction, ADM selection and implant coverage technique.

## Methods

### Questionnaire design and distribution

A national, cross-sectional online survey was developed using Google Forms and distributed electronically to UK-based consultant plastic surgeons performing breast reconstruction within the NHS. Breast surgeons who were not plastic surgery trained were excluded.

The survey encompassed the following domains: (1) unit demographics, including geographical region; (2) workload volume, including average annual number of breast reconstructions performed, proportion of immediate reconstructions that were implant-based, proportion of implant-based reconstructions performed prepectorally, and frequency of prepectoral reconstruction. Respondents who reported performing prepectoral reconstruction regularly or occasionally were directed to additional questions covering: (3) acellular dermal matrix (ADM) preference, technique of implant surface coverage (anterior surface only vs total antero-posterior surface), and factors influencing ADM selection; (4) factors influencing the decision to perform prepectoral implant reconstruction; and (5) free-text additional comments. Respondents who did not perform prepectoral reconstruction were instead asked to report their preferred reconstruction modality and reasons for not utilising the prepectoral approach.

The survey was designed to be concise, comprising approximately 11 questions, with an estimated completion time of approximately three minutes. Participation was voluntary, and all responses were fully anonymised.

The survey was disseminated via direct email to all major Plastic Surgery units across the UK by liaising with their NHS ‘workforce linkspersons’ for distribution, and to all surgeons who had available emails in the BAPRAS database. Plastic surgery departments at major hospitals were contacted by telephone to facilitate further dissemination. The survey was additionally circulated via the BAPRAS monthly bulletin (November 2025), ABS Bulletin (October 2025) and the Special Interest in Breast Surgery WhatsApp group chat. Non-respondents received up to three follow-up email reminders in accordance with the Dillman total design method to maximise response rate.^16^ The survey remained open from 5th October 2025 until 12th June 2026.

### Data collection and analysis

All responses were anonymised prior to inclusion in the final analysis. Microsoft Excel (Office 2019, Microsoft Corp., Redmond, WA) was used for preliminary data review and cleaning. Statistical analyses were performed using R version 4.5.2 (R Foundation for Statistical Computing, Vienna, Austria).

Descriptive statistics were used to summarise demographic characteristics and clinical practice data, with statistical significance being set at *p* < 0.05. Friedman’s test and pairwise Wilcoxon signed-rank tests with Bonferroni continuity correction were performed to analyse categorical differences. The Kendall W statistic was calculated as a measure of the effect size, with values ranging from 0.1–<0.3, 0.3–<0.5 and >0.5 categorised as small, moderate and large effect sizes, respectively. As these analyses were based on repeated ordinal rankings, assumptions of normality or sphericity were not required.

### Ethical considerations

No formal ethical approval was required for this survey, as it involved anonymised data collection from healthcare professionals. Participation was voluntary, and implied consent was obtained through completion of the survey.

## Results

### Demographics

20 plastic surgeons responded to the survey, of whom 19 provided geographical data. The most represented region was the East of England (n = 5), followed by London (n = 2), Scotland (n = 2), the North East (n = 2) and the North West of England (n = 2). Table 1 summarises the included plastic surgeons’ demographics.

**Table 1:** Summary of surgeon demographics.

Geographical region	N (%)
East Midlands	1 (5.3)
East of England	5 (26.3)
London	2 (10.5)
North East	2 (10.5)
North West	2 (10.5)
Northern Ireland	0 (0.0)
Scotland	2 (10.5)
South East	1 (5.3)
South West	1 (5.3)
Wales	1 (5.3)
Wessex	1 (5.3)
West Midlands	1 (5.3)
Yorkshire and the Humber	1 (5.3)

### Breast reconstruction characteristics

The reported annual volume of immediate breast reconstructions varied, ranging from 0 to 10 to 51–75 cases per year. The most frequently reported categories were 0–10 cases (n = 5) and 21–30 cases (n = 5) per annum. Table 2 summarises the reported annual immediate breast reconstruction volume.

**Table 2:** Average number of immediate breast reconstructions per annum.

Average number of immediate breast reconstruction per annum	N (%)
0–10	5 (25.0)
11–20	2 (10.0)
21–30	5 (25.0)
31–40	2 (10.0)
41–50	2 (10.0)
51–75	4 (20.0)
>75	0 (0.0)

Implant-based reconstruction represented a small proportion of immediate reconstruction practice for most respondents. Twelve surgeons (60.0%) reported that implants accounted for 0–10% of their immediate breast reconstructions. However, practice varied, with four respondents reporting that >70% of their immediate reconstructions were implant-based. Table 3 summarises reported implant use.

**Table 3:** Percentage of immediate breast reconstructions using implants.

Percentage of immediate breast reconstructions using implants (%)	N (%)
0–10	12 (60.0)
11–20	1 (5.0)
21–30	1 (5.0)
31–40	1 (5.0)
41–50	0 (0.0)
51–60	0 (0.0)
61–70	1 (5.0)
71–80	2 (10.0)
81–90	0 (0.0)
91–100	2 (10.0)

### Prepectoral implant reconstruction trends

16 of the 20 respondents reported performing prepectoral implant reconstruction and completed the prepectoral section of the questionnaire (Table 4). Eleven respondents reported high familiarity with pre-pectoral reconstruction due to regular use, while five reported moderate familiarity due to occasional use. Four respondents reported that they did not perform prepectoral reconstruction.

**Table 4:** Self-reported familiarity with prepectoral breast reconstruction.

Self-reported familiarity with pre-pectoral breast reconstruction	N (%)
High familiarity due to regular pre-pectoral reconstruction	11 (55.0)
Moderate familiarity due to occasional pre-pectoral reconstruction	5 (25.0)
Low familiarity due to no pre-pectoral breast reconstruction being performed	4 (20.0)

Among all respondents, 15 surgeons (75.0%) reported using the prepectoral plane for at least 50% of their implant-based reconstructions. The most frequently reported category was 91–100% of implant-based procedures (n = 7). Table 5 summarises the reported proportion of implant-based reconstructions performed prepectorally.

**Table 5:** Prepectoral implant reconstruction trends.

Percentage of pre-pectoral implant breast reconstructions (%)	N (%)
0–10	5 (25.0)
11–20	0 (0.0)
21–30	0 (0.0)
31–40	0 (0.0)
41–50	0 (0.0)
51–60	1 (5.0)
61–70	2 (10.0)
71–80	2 (10.0)
81–90	3 (15.0)
91–100	7 (35.0)

**Fig. 1:**
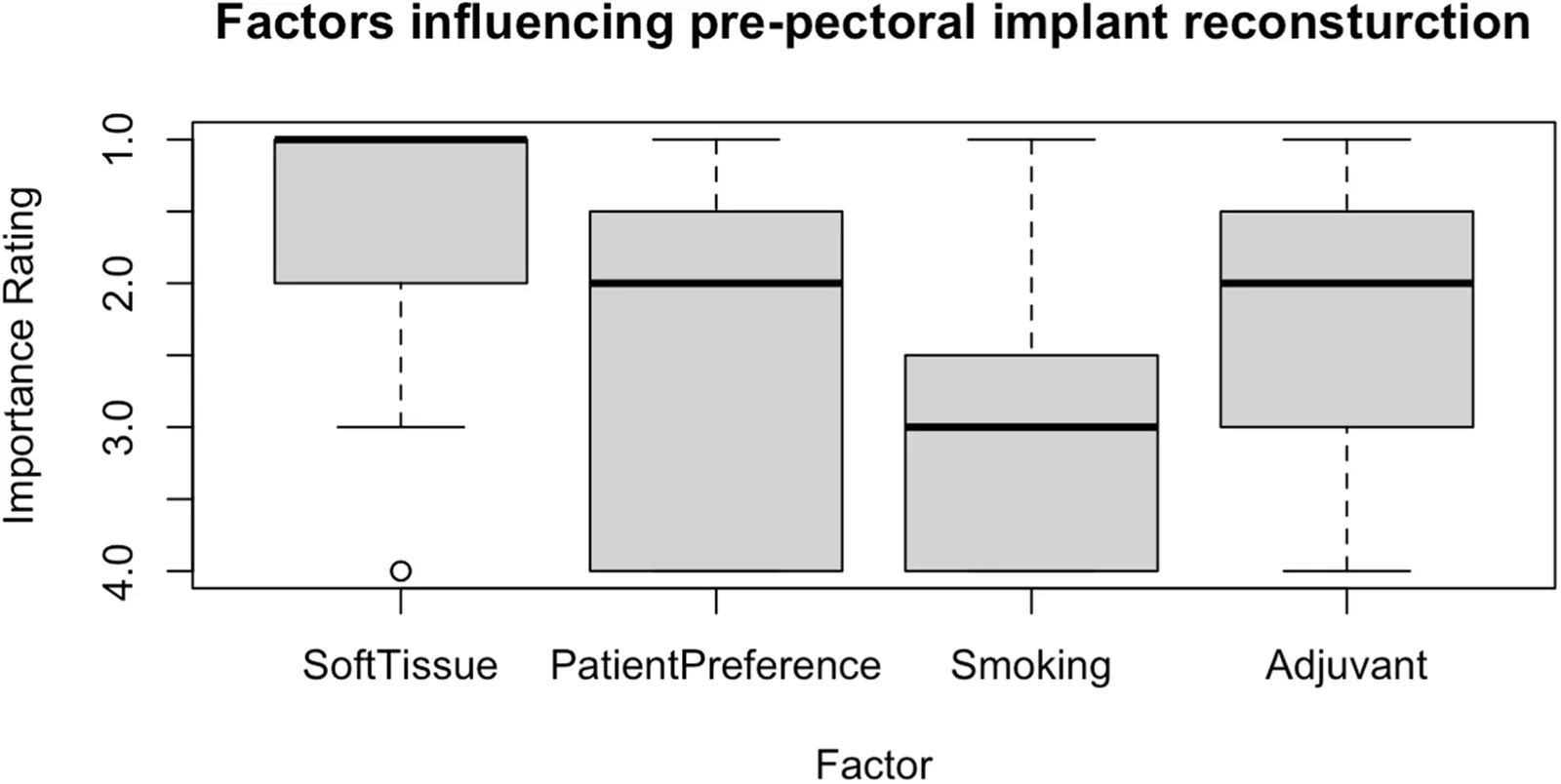
Boxplot summarising the importance ratings of factors influencing pre-pectoral implant reconstruction. The rating of 1 was given to factors of highest importance, whereas a rating of 4 was given to factors of low importance.

Three out of the four surgeons who did not perform prepectoral implant reconstructions reported carrying out only autologous free flaps procedures. The fourth surgeon performed only cosmetic breast surgery/secondary reconstruction revisions. Justifications from these surgeons for not using prepectoral reconstruction included not carrying out immediate reconstructions, lack of training with breast implants, autologous reconstructions having superior long-term outcomes and breast surgeons performing these procedures instead of plastic surgeons. 16 surgeons rated soft tissue coverage, patient preference, smoking status and planned adjuvant treatments from most important to least important in influencing the decision to perform prepectoral breast reconstructions. Fig. 1 summarises these results. A statistically significant difference was identified between ranked factors using Friedman’s test (χ²(3)=14.0; *p* < 0.01; Kendall’s W = 0.291). Pairwise comparisons with Bonferroni correction demonstrated that soft tissue coverage was ranked as significantly more important than smoking status (*p* = 0.01). No other pairwise comparisons reached statistical significance.

### ADM utilisation in prepectoral breast reconstruction

The same 16 respondents who completed the prepectoral section also provided responses regarding ADM use. Porcine ADM was the most commonly reported material, used by 14 surgeons (87.5%). Bovine ADM was reported by four surgeons (25.0%), while no respondents reported using human-derived ADM. One respondent reported using TIGR mesh rather than ADM. Table 6 summarises reported ADM and mesh use.

**Table 6:** Types of ADM used.

Types of acellular dermal matrices used	N (%)
Porcine	14 (87.5)
Bovine	4 (25.0)
Human	0 (0.0)
None but usually use TIGR mesh	1 (6.3)

With regard to implant coverage, 10 surgeons (62.5%) reported covering both the anterior and posterior implant surfaces, while five (31.3%) reported covering the anterior surface alone. The remaining respondent indicated that implant coverage depended on the type of ADM used.

**Fig. 2:**
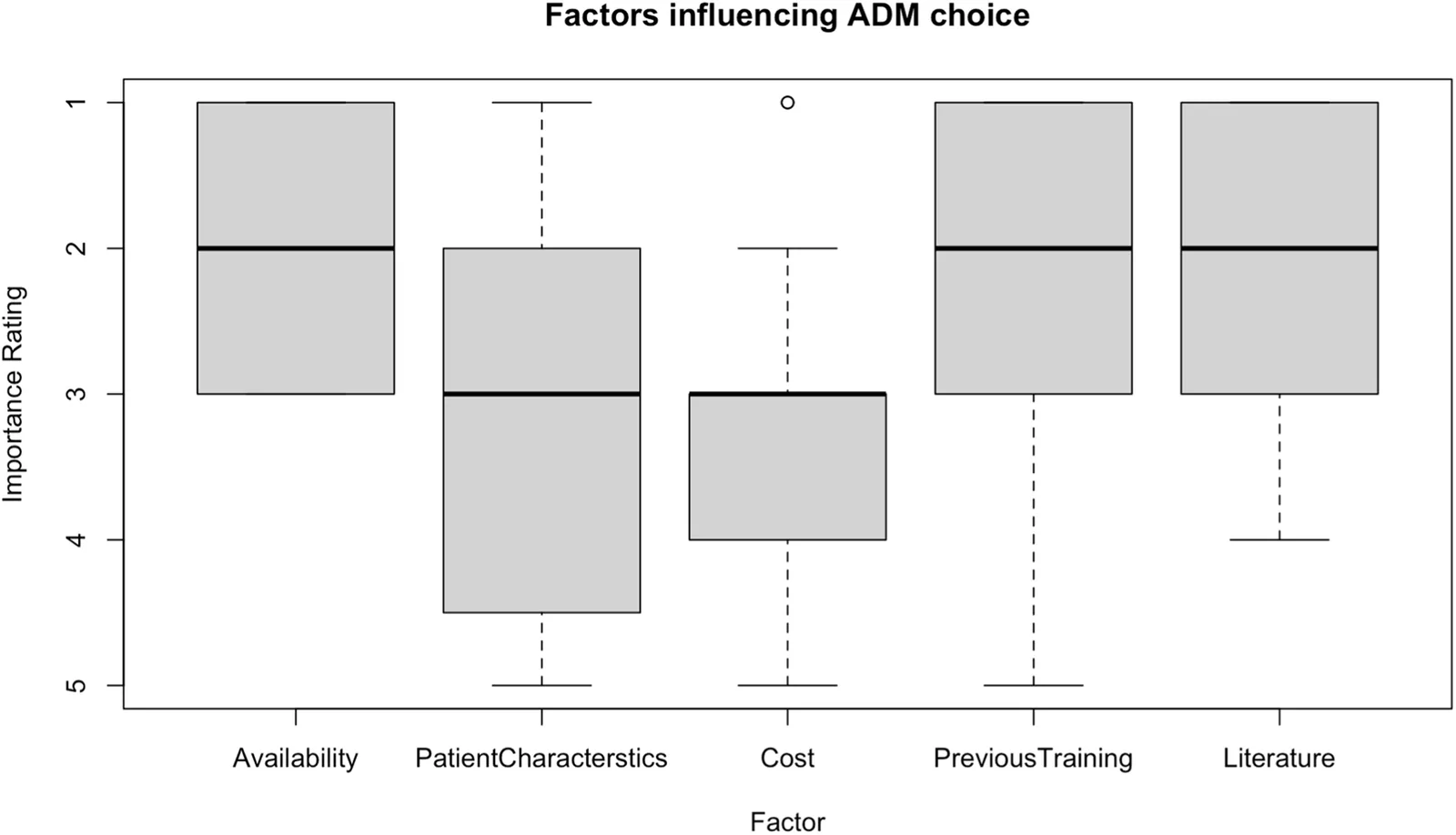
Boxplot summarising the surgeons’ ratings of factors that influence their ADM choice. A score of 1 represents the factor is of high importance, and a score of 5 demonstrates the factor is of little importance.

ADM availability, cost, previous training or exposure, literature recommendations and patient characteristics were rated from most to least important in influencing ADM selection. Friedman’s test demonstrated a statistically significant difference between ranked factors (χ²(4)=13.3; *p* = 0.01; Kendall’s W = 0.207). Pairwise Wilcoxon signed-rank testing with Bonferroni correction identified ADM availability as a significant factor in ADM selection (*p* = 0.022). No other pairwise comparisons reached statistical significance after correction. Fig. 2 summarises the factors influencing ADM choice.

### Qualitative analysis of free-text comments

Eight surgeons provided additional free-text comments regarding their reasons for performing prepectoral reconstruction and their choice of ADMs. Four main themes were identified:

• Plastic surgeon workload: Several surgeons noted that they have observed a trend of decreasing immediate and delayed breast reconstructions using implants, due to breast surgeons taking on more of this workload and being less keen on combining operations with other specialties.
• Braxon ADM: Several responses noted that Braxon ADM was straightforward to use, due to the minimal preparation required and low rate of associated skin reactions.
• Individual patient factors: Some surgeons highlighted the incredibly individualistic nature of breast reconstruction, citing a variety of patient factors such as athleticism, previous surgeries, and previous radiation therapy that subsequently influenced the patients’ treatments.
• Surgical outcomes: Several surgeons remarked that additional factors such as patient quality of life and implant loss were significant factors to consider when choosing pre-pectoral and implant surgery.

## Discussion

### Principal findings

To our knowledge, there has been no survey data on pre-pectoral implant-based breast reconstruction practice among UK plastic surgeons. Marks et al. (2020).^17^ performed a similar survey evaluating contemporary trends of pre-pectoral implant-based breast reconstruction, amongst US plastic surgeons. Mylvaganam et al. (2017).^18^ demonstrated that implant-based breast reconstruction practices in the United Kingdom remain highly variable, but did not address pre-pectoral reconstruction. They, subsequently reported variable adherence to best-practice guidelines for implant-based reconstruction.^19^ The present survey adds to this literature by describing reported pre-pectoral practice among a respondent group of UK consultant plastic surgeons. The rationale for this study stemmed from the perception that an increasing share of implant-based breast reconstructions are performed by non-plastic surgeons, underscoring the need to specifically evaluate the preferences of plastic surgeons

Three main findings emerged from this survey: (1) implant-based reconstruction formed a relatively small proportion of immediate breast reconstruction practice for most respondents; (2) among the respondents performing implant-based reconstruction, the pre-pectoral pocket was more commonly used than other techniques; and (3) there was variation in ADM selection and implant coverage technique. Free-text comments also raised the possibility of a shift in implant-based reconstruction workload away from plastic surgeons and towards breast and oncoplastic surgeons.

### Implant-based workload among plastic surgeons

The free-text comments suggested that some respondents had observed a reduction in implant-based reconstruction within their practice, with breast surgeons undertaking an increasing proportion of this workload, consistent with previous UK data. For example, Mylvaganam et al. (2017).^18^ reported a higher median number of breast surgeons carrying out implant-based breast reconstruction compared to plastic surgeons, with a significant increase since 2002.^20^ National increases in breast reconstruction rates have also been attributed to increased activity within established breast units.^21^ Changes in specialist breast surgery training and the development of oncoplastic breast surgery may therefore have contributed to changing patterns of service delivery.^22^

Although this survey cannot quantify national workforce changes, the findings raise relevant questions regarding training exposure, operative experience and outcome monitoring across plastic surgeon-led, breast surgeon-led and combined multidisciplinary models of implant-based reconstruction. This is particularly relevant if plastic surgery trainees have reduced exposure to implant-based reconstruction while continuing to be expected to understand and manage complications related to these procedures.

### Pre-pectoral reconstruction

Although implant-based reconstruction represented a small proportion of immediate reconstruction practice for most respondents, the pre-pectoral plane was commonly used by those performing implant reconstruction. Overall, a third of respondents reported using the pre-pectoral pocket in most of their implant-based reconstructions. This is consistent with reports from the USA suggesting increasing adoption of pre-pectoral reconstruction.^17^^,^^23^

Soft tissue coverage was ranked as the most important factor influencing the decision to perform pre-pectoral reconstruction and was rated significantly more important than smoking status. This is clinically plausible, given that pre-pectoral reconstruction relies on adequate mastectomy flap thickness and vascularity in the absence of pectoralis muscle coverage. However, no other pairwise comparisons reached statistical significance, and the small sample size limits the strength of inference.

The comparative evidence for prepectoral versus subpectoral reconstruction remains mixed. Some studies report broadly comparable complication rates between the two planes, with widely accepted advantages of prepectoral reconstruction including reduced animation deformity and improved chest wall morbidity.^24^, ^25^, ^26^, ^27^, ^28^ Conversely, other reports have identified increased risks of surgical site infection, seroma or the need for secondary fat grafting to address rippling in selected cohorts.^26^^,^^28^ These findings support the view that pre-pectoral reconstruction should not be considered a universal replacement for subpectoral reconstruction, but rather a technique whose suitability more critically depends on patient factors, mastectomy flap quality, adjuvant treatment plans and surgeon experience.

### ADM choice and implant coverage

Porcine ADM was the most frequently reported ADM type in this survey, while, as expected, no respondents reported using human-derived ADM as it is not licensed in the EU. This contrasts with the findings of Marks et al. (2020), in which human ADM was the most commonly used among equivalent respondents in the United States.^17^ Additional reasons for these differences include product availability, procurement, cost and local institutional practice between healthcare systems.

In this survey, ADM availability emerged as an important factor influencing whether a porcine or bovine product was used. Previous training or exposure and published literature were also reported as relevant considerations, although availability was the only factor that reached statistical significance in pairwise testing after correction. Current evidence comparing ADM materials remains limited, with available reviews suggesting broadly comparable implant loss rates across human, porcine and bovine ADM, although complication profiles may vary between products.^29^^,^^30^ Accordingly, the findings of this survey suggest that ADM selection in UK practice may be influenced not only by published evidence, but also by local availability and surgeon familiarity.

Variation was also seen in implant coverage technique. Most respondents reported covering both the anterior and posterior implant surfaces, while a smaller proportion used anterior coverage alone. Several free-text comments suggested that complete coverage was related to the use of Braxon ADM, reflecting the design and handling characteristics of this product. At present, the optimal extent of ADM coverage in pre-pectoral reconstruction remains unclear, and comparative evidence is insufficient to recommend one coverage strategy over another.^31^

### Limitations

Several limitations must be acknowledged. The sample size was small, with 20 respondents overall and 16 completing the pre-pectoral and ADM sections of the questionnaire. The findings should therefore be interpreted as a descriptive snapshot of practice among respondents rather than as a definitive representation of national practice. A formal response rate could not be calculated because the survey was disseminated through multiple overlapping channels, including professional networks, bulletins and direct emails. This introduces the possibility of selection bias, particularly as surgeons with an interest in pre-pectoral reconstruction may have been more likely to respond. Additionally, the denominator of the number of plastic surgeons performing breast reconstruction is difficult to ascertain.

The survey relied on self-reported practice and is therefore subject to recall and reporting bias. The qualitative findings were based on free-text comments from only eight respondents and should be interpreted as exploratory. As such, the study cannot describe overall UK implant-based reconstruction practice across all specialities.

Finally, the survey was open over a prolonged period, during which clinical practice may have evolved. Despite these limitations, the findings provide useful contemporary insight into reported pre-pectoral implant-based reconstruction practice among UK plastic surgeon respondents and make no claims about capturing the perspectives of breast and oncoplastic surgeons, who may perform a substantial proportion of implant-based reconstruction in the UK.

## Conclusion

This survey identified wide variation in prepectoral implant-based breast reconstruction practice among responding specialist UK consultant plastic surgeons who perform breast reconstruction, particularly in relation to ADM selection and implant coverage technique. Porcine ADM was the most commonly reported material, and local availability appeared to be an important factor influencing ADM choice. Soft tissue coverage was ranked as the most important factor influencing the decision to perform pre-pectoral reconstruction.

## Ethical approval

Not required.

## Declaration of competing interest

None declared.
